# Valproic acid-induced hyperammonemic encephalopathy: a clinical case

**DOI:** 10.1192/j.eurpsy.2022.1881

**Published:** 2022-09-01

**Authors:** M. Felizardo, C. Freitas

**Affiliations:** Centro Hospitalar de Trás-os-Montes e Alto Douro, Psiquiatria, Vila Real, Portugal

**Keywords:** hyperammonemia, encephalopathy, valproic acid

## Abstract

**Introduction:**

Valproic acid is a psychotropic drug used for several years, due to its properties as a mood stabilizer, being considered as first-line treatment for bipolar disorder. In addition to its teratogenic potential, which prevents its recommendation for the treatment of bipolar disorder in women of childbearing age, valproic acid is associated with some side effects, such as gastrointestinal symptoms, alopecia, weight gain, tremor or hepatotoxicity. Hyperammonemia is a side effect that is little described, but relatively frequent, and may progress to variable encephalopathy.

**Objectives:**

The authors describe a clinical case of a 48-year-old female patient, hospitalized due to a manic episode, who was prescribed valproic acid, in association with lorazepam and olanzapine.

**Methods:**

After three days on a dose of 1000mg of valproic acid, the patient began an acute condition of confusion, psychomotor retardation, temporal-spatial disorientation and ataxia. Infection, electrolyte disturbance and acute cerebral event were excluded. Noteworthy only hyperammonemia. Valproic acid was withdrawn and replaced by lithium, with the patient recovering from the confusional state two days later.

**Results:**

Hyperamonemic encephalopathy secondary to valproic acid was concluded. The mechanisms of valproic acid-linked hyperammonemia are not clear, although it appears to be independent of hepatotoxicity. The most studied hypotheses are related to glutamine reabsorption and serum levels carnitine in patients medicated with valproic acid.

**Conclusions:**

It is essential that there is a high level of suspicion in clinicians for this secondary effect of valproic acid, in order to adequately treat the patient who presents with acute confusional conditions, not explained by other complications.

**Disclosure:**

No significant relationships.

